# In Vivo Ultrasonographic Assessment of Bone Mineral Density and Its Impact on Semen Quality in Boars

**DOI:** 10.3390/ani15213072

**Published:** 2025-10-23

**Authors:** Miaomiao Liao, Xinyu Liu, Hengxi Wei, Li Li, Shouquan Zhang

**Affiliations:** National Engineering Research Center for Breeding Swine Industry, Guangdong Provincial Key Laboratory of Agro-Animal Genomics and Molecular Breeding, College of Animal Science, South China Agricultural University, Guangzhou 510642, China

**Keywords:** boar, bone mineral density (BMD), semen quality, 25-hydroxyvitamin D_3_ (25-OH-D_3_), reproductive hormones, breed

## Abstract

**Simple Summary:**

Bone mineral density (BMD) is a key indicator of skeletal health in boars and affects reproductive performance. In this study, 492 adult and 208 replacement boars were assessed using quantitative ultrasound (QUS) to examine BMD and its relationship with semen quality. Additionally, 150 adult Duroc boars were randomly assigned to five groups for 90 days to evaluate supplementation effects on semen quality, reproductive hormones, and bone metabolism. Results showed that BMD was influenced by breed and age and correlated with sperm abnormality rates. Supplementation with 250 μg 25-OH-D_3_ improved sperm motility, reduced abnormalities, and increased reproductive hormone and osteocalcin levels. These findings suggest that 25-OH-D_3_ supplementation can effectively enhance boar reproductive performance and bone health.

**Abstract:**

Bone mineral density (BMD) is a key indicator of skeletal health in boars that influences their reproductive performance. Systematic research on the relationship between BMD and semen quality in adult boars of different breeds and ages is scarce. This study used quantitative ultrasound (QUS) technology to measure BMD in 492 adult and 208 replacement boars. The boars were divided into four equal groups based on descending BMD rankings to analyze correlations with semen quality. Simultaneously, a 25-hydroxyvitamin D_3_ (25-OH-D_3_) intervention trial was conducted on 150 adult Duroc boars. A control group and four dose groups (50 μg, 125 μg, 200 μg, and 250 μg) were established. After 90 days, the boars’ semen quality, reproductive hormone levels, and bone metabolism indicators were evaluated. The results showed no significant differences in BMD between adult and replacement boars. However, adult Landrace exhibited significantly higher BMD than Duroc and Yorkshire (*p* < 0.01). Within the BMD groups, Group D boars had significantly higher rates of sperm abnormality than Groups A and B (*p* < 0.01), and this trend was consistent across breeds. The 25-OH-D_3_ intervention results indicated that the 250 μg dosage produced the optimal effect. In this group, boar semen motility significantly improved while sperm abnormality rates significantly decreased. Concurrently, levels of follicle-stimulating hormone (FSH), luteinizing hormone (LH), testosterone (T), serum osteocalcin (OC), and BMD all increased to some degree. In summary, boar BMD is significantly influenced by breed and age and is closely correlated with the rate of sperm abnormality rate. Supplementing with 250 μg of 25-OH-D_3_ effectively enhances reproductive hormone secretion, improves semen quality, and promotes bone formation. This demonstrates its potential value in breeding and nutritional regulation applications.

## 1. Introduction

Bone mineral density (BMD), as a key indicator for measuring bone mass and determining the severity of osteoporosis, is defined as the mineral content per unit volume of bone [1]. It not only accurately predicts fracture risk [2] but also holds significant importance for boar breeding production. Research indicates that low BMD is a primary cause of leg weakness in pigs [3]. Fractured pigs are typically culled due to the difficulty of healing fractures. Even if fractured pigs are not culled, their reproductive performance significantly declines [4]. Statistics show that the annual culling rate for boars is approximately 30%, with one-third of these culls attributed to foot and limb disorders [5]. Boars, characterized by heavy weight, limited mobility, and intensive housing, are particularly susceptible to limb and hoof disorders. Poor bone quality is the primary cause of hoof diseases and leg weakness, severely shortening their productive lifespan. Therefore, early detection and prevention of hoof and limb diseases are crucial for extending the service life of breeding stock and enhancing production efficiency.

Currently, various methods are used to assess leg and hoof health in pigs, including leg scoring, gait scoring, bone mineral content, osteomalacia scoring, and biceps femoris length [6,7,8,9,10]. However, leg and gait scoring rely heavily on operator experience and are highly subjective. Dual-energy X-ray absorptiometry (DXA), while commonly used for BMD measurement, is costly, difficult to apply in vivo, and may compromise animal welfare [11]. Therefore, developing novel, non-invasive, and objective methods for measuring animal BMD is essential. Quantitative ultrasound (QUS) has been validated as a low-cost alternative to DXA for measuring BMD and assessing fracture risk [12,13]. It offers advantages such as rapid sample data collection and no adverse health effects on animals, making it more suitable for practical production settings.

The association between BMD and reproductive hormones provides clues for investigating its relationship with boar semen quality. Studies indicate that testosterone (T) secretion is positively correlated with BMD, and infertile males exhibit significantly lower T levels than fertile males [14]. Bone tissue promotes T synthesis by secreting osteocalcin (OC), thereby regulating male fertility. Conversely, T facilitates germ cell maturation and inhibits apoptosis [15]. Furthermore, OC participates in regulating glucose metabolism, muscle mass, brain development and function, and parasympathetic tone, suggesting multi-organ connections involving bone [16,17]. Szulc et al. further demonstrated that individuals with higher levels of uncarboxylated osteocalcin (ucOC) released by osteoclasts exhibited significantly lower BMD compared to those with lower levels [18]. These studies suggest that BMD is partially correlated with reproductive capacity. In boar production, individuals with low BMD are more prone to limb and hoof disorders, leading to restricted mobility and feed intake, which in turn affects semen quality and yield. Therefore, one focus of this study is to measure boar BMD using an ultrasound BMD device and investigate the association between BMD and semen quality.

Vitamin D_3_ (VD_3_) is synthesized in the skin from 7-dehydrocholesterol upon exposure to ultraviolet (UV) light and subsequently undergoes hydroxylation in the liver to form 25-OH-D_3_, the primary circulating form of VD. 25-OH-D_3_, a fat-soluble nutrient, plays a pivotal role in male reproductive function, as its metabolizing enzymes and vitamin D receptors (VDR) are expressed throughout the male reproductive tract and in germ cells [19]. These findings suggest that 25-OH-D_3_ may directly influence spermatogenesis and overall reproductive performance in males. Research indicates that 25-OH-D_3_ modulates sperm function by influencing sperm motility and acrosome reaction through Ca^2+^-dependent mechanisms [20]. Appropriate supplementation of 25-OH-D_3_ in boar diets increases fructose and Ca^2+^ concentrations in semen, elevates serum estradiol and testosterone levels, and promotes reproductive tissue development [21]. Furthermore, studies indicate it enhances seminal plasma antioxidant capacity and reduces cortisol’s suppression of testosterone secretion, thereby improving boar reproductive performance [22,23].

## 2. Materials and Methods

### 2.1. Ethical Statement

All animals used in this study were raised in accordance with Chinese swine regulations and approved by the Ethics Committee of South China Agricultural University (Approval No.: 2022G021).

### 2.2. Experimental Animals

All 700 boars originated from a boar breeding farm in Guangdong. These boars comprised three breeds: Duroc, Landrace, and Yorkshire. Additionally, 492 boars with an average age of 24.28 months (range: 22.03–28.60 months) were defined as adult boars, while 208 boars with an average age of 10.60 months (range: 8.6–11.53 months) were defined as replacement boars. All experimental boars were confirmed to be in healthy condition before and after to the study, with no diseases, normal mobility, regular feeding, and participation in routine production activities. Environmental variables within the boar house were controlled via air conditioning. Temperature and humidity were maintained at 20–22 °C and 65–75%, respectively. Boars were housed in individual stalls. All boars received identical feed and water; mature boars were fed 2.5 kg of feed daily, while replacement boars received 2 kg daily. Feed ingredients and nutritional composition are detailed in Table 1.

### 2.3. BMD Measurement Method

BMD of the hind limbs of boars was measured using an ultrasonic BMD analyzer (Nanjing Kojin Industrial Co., Ltd., Nanjing, China, model OS TEOKJ7000+), with all measurements performed by the same operator. BMD was quantified as sound velocity (SOS), which reflects the speed at which ultrasound waves propagate through bone and is positively correlated with bone density and structural integrity (m/s). To measure BMD, the bone mineral density probe was placed on the medial metatarsal bone of the boar’s hind limb. Each measurement lasted over 30 s and was repeated three times. The recorded value for each boar was the average of the three measurements.

### 2.4. Grouping of Boars

#### 2.4.1. Grouping of Adult Boars

Since no established classification standard for BMD in boars currently exists and the sample size in this study was insufficient to develop a new reference, adult boars were ranked according to their measured BMD values, from highest to lowest, and divided into four groups: Group A (SOS > 4335.21 m/s), Group B (3951.81 m/s ≤ SOS ≤ 4335.21 m/s), Group C (3664.26 m/s ≤ SOS < 3951.81 m/s), and Group D (SOS < 3664.26 m/s).

#### 2.4.2. Grouping of 25-OH-D_3_ Test Boars

A total of 150 Duroc boars aged 1.5 years with BMD values below 4000 m/s were selected from Groups B, C, and D. The boars were randomly allocated to five groups (*n* = 30 per group), including one control and four treatment groups receiving dietary 25-OH-D_3_ supplementation at doses of 0 μg, 50 μg, 125 μg, 200 μg, and 250 μg, respectively. The supplement was incorporated into the feed and administered for a period of 90 days. This experimental design allowed assessment of the effects of graded 25-OH-D_3_ supplementation on semen quality, reproductive hormone profiles, and bone metabolism in boars.

### 2.5. Semen Quality Analysis

Each adult boar underwent semen collection once every four days, for a total of three sessions during the experimental period. Each sample was analyzed within five minutes of collection. Semen data from replacement boars were excluded from analysis, as these animals were still undergoing training, and their semen quality was highly variable and did not meet production standards. Due to manpower and time constraints, no semen preliminary collections were performed; however, all included boars were suitable for practical production both before and after the study, indicating normal semen quality. All collections and assessments were conducted by fixed, experienced personnel to ensure accuracy, reliability, and minimal bias. Semen collection was performed in accordance with the Technical Specification for Production and Preservation of Room-Temperature Semen from Boar (GB/T 25172-2020) [24]. Semen volume was determined by weighing each ejaculate using an electronic balance (Magapor S.A., Zaragoza, Spain, model S3R-6KD), and the weight was converted to volume assuming a density of 1 g/mL; sperm motility, sperm abnormal rate, and semen concentration were assessed using the Magapor Gesipor 3.0 CASA system (Magapor S.A., Zaragoza, Spain, model Magavision). For each semen sample, at least 500 spermatozoa were examined microscopically. Sperm motility was determined based on movement characteristics, while sperm morphology was assessed according to standard evaluation criteria. Semen concentration was calculated and expressed as ×10^8^ sperm/mL. All semen collections and analyses were conducted by the same trained personnel to ensure methodological consistency, accuracy, and reproducibility.

### 2.6. Blood and Semen Collection and Reproductive Parameter Assessment in the 25-OH-D_3_ Experiment

On days 1 and 90 of the trial, blood samples were collected from the boars after an 8 h fasting period. In parallel, semen samples were also collected before and after the supplementation period to evaluate reproductive parameters. To minimize stress and ensure animal welfare, trained personnel performed all procedures in strict accordance with institutional and national guidelines for the care and use of experimental animals. During collection, each boar was calmly restrained in a standard handling crate, and 5 mL of blood was drawn from the hind leg vein without the use of anticoagulants or other interventions. The blood samples were allowed to stand at room temperature for 1–2 h, followed by centrifugation at 1000 rpm for 10 min. The supernatant was collected and stored at −20 °C. Serum samples were subsequently sent to Shanghai Shenggong Biotech Co., Ltd. (Shanghai Shenggong Biotech Co., Ltd., Shanghai, China, model ml002384, ml002326, ml705604 and ml002339) for analysis using a microplate reader (ELISA).

### 2.7. Statistical Analysis

#### 2.7.1. Calculation of Adult Boar Semen Data

All statistical analyses for this section were performed in R 4.5.1. Data normality was assessed using the Shapiro–Wilk test, and homogeneity of variance was evaluated using the Levene (Brown–Forsythe) test. Continuous variables are expressed as mean ± standard deviation (mean ± SD). All tests were two-tailed, with a significance threshold set at *p* < 0.05.

For all boar samples, a linear model (ANCOVA) was fitted with BMD as the dependent variable and age, breed, and their interaction as independent variables. *p*-values for the main effects of age, breed, and interaction terms were analyzed based on Type-III variance analysis. For adult boar semen data, linear mixed-effects models were fitted for volume, density, motility, and abnormal sperm rate. Type-III ANOVA with Satterthwaite approximation yielded *p*-values. Semen samples were grouped into four groups (A–D) based on descending BMD. One-way ANOVA compared intergroup differences, while intra-breed comparisons within groups A–D also employed ANOVA. For BMD comparisons (adult boars vs. replacement boars), Welch’s *t*-test was used between age groups. Within adult boars and replacement boars, ANOVA was used to compare different breeds. For each breed, Welch’s *t*-test was used to compare adult boars and replacement boars.

The formula is:

$$BD_{i}=\beta_{0}\;+\;\beta_{Age\;in\;months}\cdot {Age\;in\;months}_{i}\;+\;\beta_{Breed}\cdot {Breed}_{i}+\beta_{Interaction\;effect}\;\cdot \left({Age\;in\;months}_{i}\times {Breed}_{i}\right)+\varepsilon_{i}.$$

$BD_{i}\;$ represents the BMD of the i^th^ boar; $\beta_{0}$ is the constant term (intercept); $\beta_{Age\;in\;months}$ is the effect coefficient for age in months; $\beta_{Breed}$ is the effect coefficient for breed; $\beta_{Interaction\;effect}$ is the coefficient for the interaction effect between age and breed; and $\varepsilon_{i}$ is the error term, which follows a normal distribution.

$$Y_{ij}=\beta_{0}+\beta_{Breed}\cdot {Breed}_{i}+\beta_{\mathrm{BD}}\cdot {\mathrm{BD}}_{i}+\beta_{Interaction\;effect}\cdot \left({Breed}_{i}\times {\mathrm{BD}}_{i}\right)+u_{i}+\varepsilon_{ij}.$$

$Y_{ij}$ represents the j^th^ observed semen quality index value (e.g., semen volume, semen density, etc.) for the ith adult boar; $\beta_{0}$ is the constant term (intercept); $\beta_{Breed}$ is the effect coefficient for breed; ${\mathrm{BD}}_{i}$ denotes the BMD of the ith boar; $\beta_{\mathrm{BD}}$ is the effect coefficient for BMD; $\beta_{Interaction\;effect}$ is the coefficient for the interaction effect between breed and BMD; $u_{i}$ is the random effect for each boar, following a normal distribution $N\left(0,\sigma_{u}^{2}\right)$; and $\varepsilon_{ij}$ is the error term, following a normal distribution $N\left(0,\sigma_{u}^{2}\right)$.

#### 2.7.2. Calculation of 25-OH-D_3_ Test Data

Statistical analysis of this section’s data was performed using GraphPad Prism 9.0 software. Data are expressed as mean ± SEM. All comparisons employed independent samples *t*-tests.

## 3. Results

### 3.1. Relationship Between Boar Semen Quality and BMD

#### 3.1.1. Factors Affecting Boar BMD and Semen Quality

The calculation results indicate that the main effect of age in months is significant (*p* = 0.0054), suggesting a stable linear relationship between age in months and BMD. The main impact of breed is not significant (*p* = 0.234), indicating that the average BMD differences among breeds are not pronounced when interaction is not considered. The interaction between age in months and breed shows marginal significance (*p* = 0.0888, at the 10% significance level), suggesting that the slope of the “age–BMD” relationship may vary slightly among breeds (Table 2). In the analysis of adult boar semen samples, the overall *p*-values for breed effects, BMD effects, and their interaction were mainly above 0.10 for the four parameters—semen volume, semen density, sperm motility, and sperm abnormality rate—indicating no statistically significant differences. The only significant effect observed was that of BMD on sperm abnormality rate (*p* = 0.00001), indicating a significant correlation between BMD changes and Sperm abnormality rate after controlling for repeated measurements within individuals (Table 3).

#### 3.1.2. Comparison and Grouping of BMD in Adult Boars and Replacement Boars

We compared the BMD of adult boars and replacement boars, with results shown in Table 4. The average BMD for adult boars was 4141.189 m/s, while that for replacement boars was 4108.226 m/s. The BMD values were relatively close, showing no significant difference at the commonly used 5% significance level but considered marginally significant at the 10% level. This discrepancy may stem from the fact that replacement boars are in a phase of rapid skeletal growth and mineralization, resulting in more pronounced BMD variations among individuals. In contrast, adult boars have completed skeletal development, exhibiting stable peak BMD values and consequently smaller inter-group BMD differences.

#### 3.1.3. Comparison of BMD Among Different Breeds of Boars

To investigate the influence of breed on BMD, we grouped and compared the BMD of adult boars and replacement boars by breed. We found that among adult boars, the BMD of Duroc and Landrace boars was significantly lower than that of Yorkshire (*p* < 0.05) (Table 5), but this phenomenon did not occur in replacement boars. Subsequently, we compared BMD between adult and replacement boars within the same breed. Results indicated that in Landrace, adult boars exhibited significantly higher BMD than replacement boars (*p* < 0.05). In Duroc, BMD between adult and replacement boars was similar, with differences marginally significant at the 10% significance level but not at the commonly used 5% level (Table 6).

#### 3.1.4. Comparison of Semen Quality Among Adult Boars in Different BMD Groups

First, we compared semen quality among boars in different BMD groups. Results showed that the sperm abnormality rate in Group D was significantly higher than in Groups A and B (*p* < 0.05) (Table 7). Subsequently, we compared semen quality among boars of the same breed but different BMD groups. Significant differences in sperm abnormality rates were also observed between BMD groups (Table 8). Specifically, among adult Landrace boars, significant differences existed among Groups A, B, and D, with Group C significantly higher than Group A (*p* < 0.05); among adult Yorkshire, no significant difference was observed between Groups A and B, while Groups A and B were significantly lower than Groups C and D (*p* < 0.05). Notably, across all three breeds, sperm abnormality rates showed a trend of increasing with decreasing BMD.

### 3.2. Effects of 25-OH-D_3_ Supplementation in Feed on Boar Reproductive Performance

#### 3.2.1. Effects of Different Doses of 25-OH-D_3_ on Boar Semen Quality

**Figure 1:**
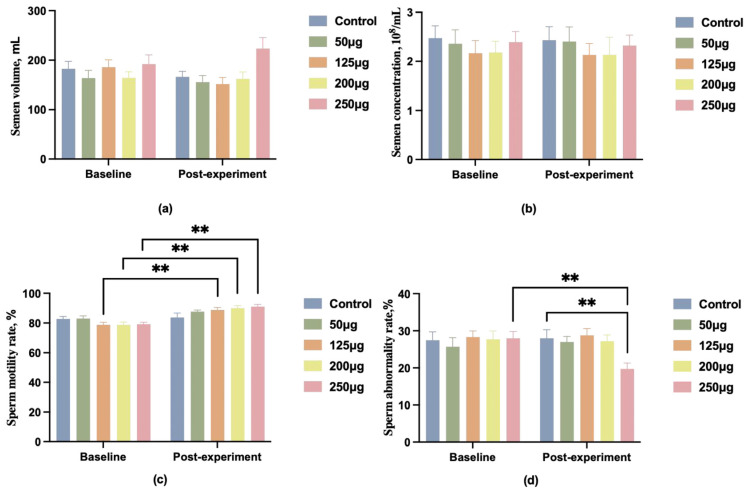
A comparison of boar semen quality before and after feeding different doses of 25-OH-D_3_. (**a**) Comparison of boar semen volume; (**b**) comparison of boar semen concentration; (**c**) comparison of boar sperm motility; (**d**) comparison of boar sperm abnormal morphology rate. ** indicates a highly significant difference (*p* < 0.01), and no symbol indicates no significant difference.

We administered different doses of 25-OH-D_3_ (0 μg, 50 μg, 125 μg, 200 μg, 250 μg) to 150 1.5-year-old Duroc boars with BMD below 4000. Data indicated no significant differences in semen quality among groups before the experiment (*p* > 0.05). Post-experiment, changes in semen volume and concentration showed no marked differences between groups. The 250 μg dose group exhibited a slight upward trend in semen volume, yet this did not differ significantly from other groups (Figure 1a,b). Intragroup longitudinal analysis revealed significantly increased sperm motility in the 125 μg, 200 μg, and 250 μg groups compared to pre-experimental levels (*p* < 0.01) (Figure 1c). The 250 μg group also exhibited a significant reduction in sperm abnormality rate compared to pre-experimental levels (*p* < 0.01) (Figure 1d).

#### 3.2.2. Effects of Different Doses of 25-OH-D_3_ on Reproductive Hormones in Boars

**Figure 2:**
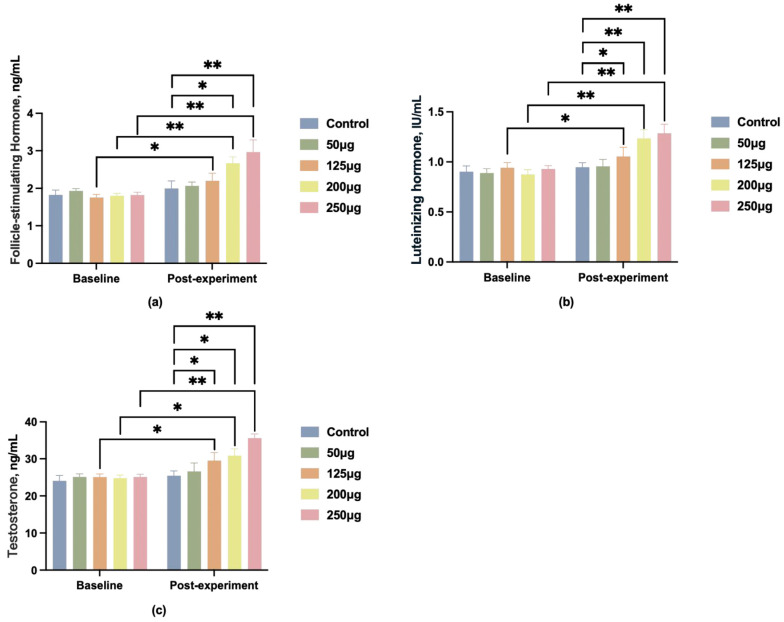
Changes in reproductive hormone levels in boar blood after feeding different doses of 25-OH-D_3_. (**a**) Changes in follicle-stimulating hormone (FSH) levels; (**b**) changes in luteinizing hormone (LH) levels; (**c**) changes in testosterone (T) levels. * indicates significant difference (*p* < 0.05), ** indicates highly significant difference (*p* < 0.01), and no label indicates no significant difference.

To better observe the effects of different doses of 25-OH-D_3_ on boar reproductive performance, blood samples were collected from the hind leg veins of the boars on day 90 of the trial. Serum was separated by centrifugation, and the levels of follicle-stimulating hormone (FSH), luteinizing hormone (LH), and testosterone (T) in the serum were measured. Compared with the control group, all four treatment groups exhibited varying degrees of elevation in FSH, LH, and T levels, with the most pronounced changes observed in the 250 μg dose group (Figure 2).

#### 3.2.3. Effects of Different Doses of 25-OH-D_3_ on Bone Mass in Boars

**Figure 3:**
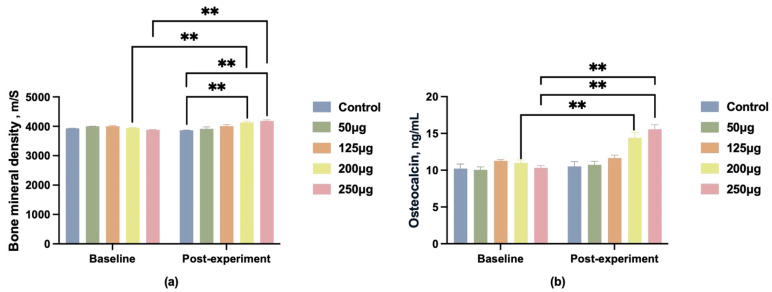
Changes in BMD and OC levels in male pigs after feeding different doses of 25-OH-D_3_. (**a**) Changes in bone mineral density (BMD); (**b**) changes in osteocalcin (OC) levels. ** indicates significant difference (*p* < 0.01), and no label indicates no significant difference.

In addition to semen quality, we collected data on boar BMD before and after the experiment, as well as OC levels in boar blood post-experiment. Before feeding, the BMD differences between groups were not statistically significant. After feeding, only the 200 μg and 250 μg dose groups showed a significant increase in bone density (*p* < 0.01) (Figure 3a), with the 250 μg dose group exhibiting a more pronounced upward trend (*p* < 0.01).

OC serves as a crucial biomarker for skeletal quality. Blood samples were collected post-experiment for OC content analysis. Following dietary intervention, serum OC levels in the 125 μg, 200 μg, and 250 μg groups were significantly higher than those of the control group (*p* < 0.01), with the 250 μg group demonstrating the most pronounced effect (Figure 3b).

## 4. Discussion

### 4.1. Differences in Bone Mineral Density (BMD) Measurement Methods

Different BMD measurement methods vary in their principles and dimensional resolution, which may influence the results. DXA is a commonly used technique for assessing BMD. As a two-dimensional projection method, it is susceptible to bone size and soft tissue effects and cannot comprehensively reflect bone microarchitecture [12]. Quantitative computed tomography (QCT) provides three-dimensional volumetric BMD information and can differentiate cortical from trabecular bone; however, it is costly, involves high radiation exposure, and is not suitable for in vivo or repeated measurements [13]. In contrast, QUS evaluates bone density, elasticity, and microstructural properties through SOS and broadband ultrasound attenuation. QUS is radiation-free, portable, and repeatable, making it particularly suitable for on-site and longitudinal studies in experimental animals such as pigs [24]. Overall, QUS offers significant advantages in safety, flexibility, and comprehensive assessment of bone quality, making it a valuable tool for animal bone research.

### 4.2. Relationship Between BMD and Boar Breeds

In this experiment, the BMD of 492 adult boars and 208 replacement boars. Results showed that BMD exhibited breed differences in adult boars, whereas no such phenomenon was observed in replacement boars. This aligns with previous research conclusions indicating significant BMD variations among different animal breeds [25,26,27].

Among adult boars, Yorkshire individuals exhibited significantly higher BMD compared with Duroc and Landrace. This phenomenon may be related to the premature culling of Durocs and Landraces with low BMD due to leg disorders. Furthermore, since only three breeds were included in this study and only Yorkshires showed significant differences in BMD between adult and replacement boars, it is speculated that this situation may be specific to certain breeds and related to differences in BMD growth rates across different periods among breeds. Damaziak et al. observed period-specific variations in BMD growth rates among three chicken breeds, with some breeds exhibiting gradual increases while others showed significant increases only at 21, 35, or 56 days of age [26]. Zotti et al. found that among beef cattle, only Charolais exhibited significant BMD differences compared to three other breeds [27]; Kumar et al. also demonstrated differences in bone mass, growth rate, mineralization rate, and BMD across three dog breeds at different developmental stages [28].

### 4.3. Relationship Between BMD and Boar Semen Quality

In our study, we found significant differences in sperm abnormality rates among boars with different BMD levels, which may be directly related to reduced bone mass. Bone tissue can promote testosterone synthesis in testicular interstitial cells through OC, which in turn supports germ cell maturation [15]. As an osteogenic hormone, OC plays a key role in the bone–testicular axis [15,16,17]. Szulc et al. [18] reported that individuals with high levels of unOC (released by osteoclasts) exhibit significantly lower BMD than those with low ucOC levels [18]. Low BMD may also predispose boars to limb and hoof disorders, increasing the risk of fractures and other musculoskeletal issues [29]. In this study, all experimental boars were housed in individual stalls with concrete slatted floors. As the boars grew larger, available space decreased, leading to markedly reduced activity. Confinement in stalls with concrete flooring further exacerbated foot and leg disorders, resulting in lower semen collection frequencies and consequently higher sperm abnormality rates [30,31].

The present study suggests that VD deficiency may be an additional factor contributing to variations in sperm abnormality rates among boars with different BMD levels. VD is essential for skeletal development and function; its insufficiency impairs bone mineralization, reduces BMD, and increases fracture risk. VD also influences semen quality via OC [32], and deficiency has been associated with elevated sperm abnormality rates [33]. VD acquisition depends on both sunlight synthesis and dietary intake. Although boars can synthesize VD through sunlight exposure [34], intensive livestock management limits their access to sunlight, making them reliant on limited dietary VD sources [35]. Consequently, boars are prone to VD deficiency, and dietary VD supplementation has been shown to reduce sperm abnormality rates [36]. In this study, boars were housed in enclosed barns, preventing natural VD synthesis. Combined with limited dietary VD, this likely resulted in suboptimal VD status, which may have contributed to reduced BMD and increased sperm abnormality rates.

Several studies have suggested a link between BMD and male reproductive health. Yang et al. [37] reported that Chinese men with low BMD typically exhibited decreased sperm motility, increased sperm abnormality rates, and a tendency toward slightly lower semen concentration. Ferlin et al. [38] observed in a cohort of 5177 men that individuals with low sperm count and low testosterone frequently had reduced BMD, accompanied by metabolic abnormalities, with overall trends of decreased sperm motility and concentration. Bobjer et al. [39] also reported that subfertile men with hypogonadism had significantly lower BMD compared with men with normal testosterone levels, along with reductions across semen parameters, including sperm motility, concentration, and morphology.

In comparison, our study showed that boars with lower BMD exhibited significantly higher sperm abnormality rates, while other semen parameters, including sperm motility, concentration, and semen volume, remained unchanged. This indicates that in boars, decreased BMD primarily affects sperm morphology, with minimal impact on other semen parameters. Although interspecies differences exist, this trend aligns with human studies reporting increased sperm morphological abnormalities in individuals with low BMD, highlighting a potential link between bone health and reproductive function and suggesting that the bone–reproductive axis may operate similarly across species.

### 4.4. Effects of 25-OH-D_3_ on Boar Reproductive Performance

25-hydroxyvitamin D_3_ (25-OH-D_3_), generated by hepatic hydroxylation of vitamin D (VD), exerts its physiological actions through binding to vitamin D receptors (VDRs) expressed in germ cells, spermatozoa, interstitial cells, and epithelial cells of the male reproductive tract [40]. These receptors enable 25-OH-D_3_ to directly regulate spermatogenesis, reproductive hormone secretion, and sperm maturation [41]. In the present study, dietary supplementation with 25-OH-D_3_ at doses of 200 μg and 250 μg significantly enhanced sperm motility. This improvement may be associated with the role of 25-OH-D_3_ in promoting intestinal calcium absorption, as calcium signaling—regulated by VDR in the boar reproductive tract—facilitates sperm tail movement, activation, and capacitation [20,42].

Within the testis and kidney, 25-OH-D_3_ is further converted to its active form, 1α,25(OH)_2_D_3_, by 25-OH-D_3_ 1α-hydroxylase (CYP27B1) [43]. The active metabolite binds to VDR and forms a heterodimer with the retinoid X receptor (RXR), which translocates into the nucleus and interacts with vitamin D response elements (VDREs) in DNA [44]. Through this mechanism, it regulates the transcription of genes critical for spermatogenesis and testicular development, including steroidogenic enzymes (CYP11A1, CYP19A1), the testicular regulator INSL3, and receptors for follicle-stimulating hormone (FSHR) and luteinizing hormone (LHR), thereby promoting testosterone synthesis, maintaining Leydig and Sertoli cell function, and supporting sperm maturation [45,46].

In addition to the classical nuclear pathway, 1α,25(OH)_2_D_3_ can rapidly activate non-genomic signaling via membrane-associated VDR, engaging MAPK/ERK and PI3K/AKT cascades that regulate cell proliferation, survival, and hormone synthesis [47,48]. It also enhances sperm mitochondrial function by upregulating antioxidant enzymes such as catalase (CAT) and superoxide dismutases (SOD1/2), maintaining ATP production and improving sperm motility and flagellar activity [49].

Furthermore, 25-OH-D_3_ markedly increases serum testosterone concentrations in boars. This may occur through 1α,25(OH)_2_D_3_-induced stimulation of CYP11A1 and CYP17A1 expression in adrenal cortical cells, thereby enhancing testosterone biosynthesis [50]. Testosterone facilitates the development of germ cells, testes, and accessory sex glands [51], elevates seminal vesicle weight and secretory activity [52], and provides fructose—accounting for approximately 60% of seminal plasma—as an energy source for sperm, further promoting motility [53]. Concurrently, 25-OH-D_3_ elevates luteinizing hormone (LH) and follicle-stimulating hormone (FSH) levels, which act through the hypothalamic–pituitary–gonadal axis to stimulate spermatogenesis and androgen secretion [54].

### 4.5. Relationship Between 25-OH-D_3_ and Boar Bone Structure

25-OH-D_3_ is produced through hepatic hydroxylation of VD, exhibiting high activity and stability. It regulates calcium-phosphorus metabolism and bone growth. Calcium ions absorbed through the bloodstream enter bone tissue, achieving a dynamic calcium equilibrium via bone resorption and formation. This study demonstrated that boars supplemented with 200 μg and 250 μg of 25-OH-D_3_ exhibited significantly higher BMD than the control group. This aligns with findings by Jefferies et al. (2002) and Sugiyama et al. (2013): The former study found that 0.1 mg/kg 25-OH-D_3_ reduced the incidence and severity of porcine osteochondrosis [53], while the latter confirmed its ability to elevate serum active VD levels in castrated boars, decrease osteochondrosis incidence, and promote normal endochondral ossification [54].

25-OH-D_3_ also significantly increases serum OC levels in boars. OC is a specific indicator reflecting bone formation rate and is positively correlated with BMD; its concurrent elevation suggests increased bone formation and bone mass growth. Notably, OC not only influences bone formation but also modulates the male reproductive system. Oury et al. found that OC stimulates testosterone production in testicular interstitial cells [15], while 25-OH-D_3_ may indirectly influence testosterone levels by regulating calcium homeostasis or OC. This explains the synchronous increase in T and OC levels observed in this study following 25-OH-D_3_ supplementation. Research on the effects of 25-OH-D_3_ on OC secretion in livestock and poultry remains limited. This study demonstrates through BMD and OC data that 25-OH-D_3_ indeed enhances skeletal strength in male pigs. However, the specific mechanism by which 25-OH-D_3_ influences OC secretion requires further investigation.

## 5. Conclusions

Analysis of BMD revealed no significant age-related differences in BMD among boars aged 10–24 months. Breed differences in BMD were observed only in adult boars, and BMD levels correlated with sperm abnormality rates. The results indicate that supplementing diets with 250 μg of 25-OH-D_3_ significantly enhances boar sperm motility, reduces sperm abnormality rates, improves overall semen utilization, increases reproductive hormone secretion, and boosts reproductive performance. Concurrently, it markedly elevates boar OC secretion and tibial BMD [1].

## Figures and Tables

**Table 1:** Nutritional composition and raw material composition of boar diet.

Nutritional Composition	Ratio, %	Raw Material Composition	Ratio, %
Crude Protein	17.03	Cornmeal	45.0
Crude Fibre	2.84	Soybean Meal	17.5
Crude Ash	5.47	Broken Rice	12.5
Crude Fat	3.34	Bran	5.5
Calcium	0.90	Expanded Soybean	7.5
Total phosphorus	0.55	Beet Pulp	5.5
		Fish Meal	2.5
		Others	6.0

**Table 2:** Effects of different factors on BMD.

Items	Age in Months	Breed	Age in Months × Breed
BMD	0.0054	0.2340	0.0888

**Table 3:** Effects of different factors on semen quality.

Items	Semen Volume	Semen Concentration	Sperm Motility	Sperm Abnormality Rate
Breed	0.503	0.116	0.609	0.545
BMD	0.304	0.866	0.638	0.00001
Breed × BMD	0.700	0.125	0.667	0.736

**Table 4:** Comparison of BMD between adult boar and replacement boars.

Items	Number	Age in Months	Average Value of BMD
Adult boars	492	24.28 ± 1.85	4141.19 ± 192.16
Replacement boars	208	10.60 ± 0.60	4108.23 ± 210.30
*p*-value	0.053		

**Table 5:** Comparison of BMD of Boars Between Different Breeds.

Items/Breeds	Adult Boars			Replacement Boars		
	Duroc	Landrace	Yorkshire	Duroc	Landrace	Yorkshire
Number	217	123	152	165	12	31
Age in months	24.27 ± 1.21	26.05 ± 1.87	25.90 ± 1.93	10.11 ± 0.65	10.56 ± 0.06	10.04 ± 0.05
Average value of BMD	4114.23 ± 191.34 ^a^	4116.25 ± 196.41 ^a^	4199.86 ± 177.53 ^b^	4112.01 ± 210.54	4037.33 ± 204.54	4115.52 ± 212.99
*p*-value	0.00003			0.485		

Note: Values in the same row with different superscript letters are significantly different (*p* < 0.05). Values sharing the same letter within a row are not significantly different (*p* > 0.05).

**Table 6:** Comparison of BMD between breeds.

Items/Breeds	Duroc		Landrace		Yorkshire	
	Adult Boars	Replacement Boars	Adult Boars	Replacement Boars	Adult Boars	Replacement Boars
Number	217	165	123	12	152	31
Age in months	24.27 ± 1.21	10.11 ± 0.65	26.05 ± 1.87	10.56 ± 0.06	25.90 ± 1.93	10.04 ± 0.05
Average value of BMD	4114.23 ± 191.34	4112.01 ± 210.54	4116.25 ± 196.41	4037.33 ± 204.54	4199.86 ± 177.53	4115.52 ± 212.99
*p*-value	0.069		0.258		0.046	

**Table 7:** Comparison of semen quality of adult boars with different BMD groups.

Items/Breeds	Number	Semen Volume, mL	Semen Concentration, 10^8^/mL	Sperm Motility	Sperm Abnormality Rate
A	123	224.577 ± 83.275	2.079 ± 0.847	0.85 ± 0.08	0.264 ± 0.136 ^a^
B	123	209.213 ± 80.986	2.112 ± 0.879	0.85 ± 0.08	0.272 ± 0.147 ^a^
C	123	226.785 ± 97.382	2.156 ± 0.893	0.83 ± 0.11	0.296 ± 0.128 ^ab^
D	123	216.218 ± 100.711	2.142 ± 1.03	0.83 ± 0.09	0.309 ± 0.1272 ^b^
*p*-value		0.284	0.307	0.221	0.032

Note: Values in the same row with different superscript letters are significantly different (*p* < 0.05). Values sharing the same letter within a row are not significantly different (*p* > 0.05).

**Table 8:** Comparison of semen quality among three breeds of adult boars with different BMD groups.

Breeds	Items/Groups	A	B	C	D	*p*-Value
Duroc	Number	45	59	51	62	
	Semen volume, mL	161.72 ± 40.69	166.17 ± 34.51	165.12 ± 47.96	164.94 ± 51.24	0.809
	Semen concentration, 10^8^/mL	2.13 ± 0.87	2.46 ± 0.84	2.34 ± 0.90	2.36 ± 1.12	0.490
	Sperm motility	0.82 ± 0.09	0.83 ± 0.09	0.81 ± 0.11	0.82 ± 0.09	0.861
	Sperm abnormality rate	0.313 ± 0.14 ^a^	0.326 ± 0.143 ^a^	0.351 ± 0.107 ^b^	0.38 ± 0.131 ^c^	0.043
Landrace	Number	30	22	33	38	
	Semen volume, mL	281.56 ± 67.51	275.02 ± 74.84	277.26 ± 81.67	281.68 ± 84.91	0.957
	Semen concentration, 10^8^/mL	2.08 ± 0.61	1.94 ± 0.51	1.91 ± 0.64	2.04 ± 0.69	0.784
	Sperm motility	0.85 ± 0.05	0.82 ± 0.08	0.80 ± 0.12	0.82 ± 0.08	0.081
	Sperm abnormality rate	0.208 ± 0.149 ^a^	0.247 ± 0.113 ^b^	0.274 ± 0.122 ^bc^	0.3 ± 0.104 ^c^	0.029
Yorkshire	Number	54	43	30	25	
	Semen volume, mL	251.46 ± 70.17	254.06 ± 76.36	261.62 ± 77.75	243.96 ± 81.72	0.991
	Semen concentration, 10^8^/mL	2.00 ± 0.64	2.13 ± 0.74	2.43 ± 1.08	1.97 ± 0.67	0.303
	Sperm motility	0.87 ± 0.07	0.88 ± 0.06	0.89 ± 0.06	0.86 ± 0.10	0.966
	Sperm abnormality rate	0.192 ± 0.116 ^a^	0.21 ± 0.111 ^a^	0.234 ± 0.1 ^b^	0.264 ± 0.116 ^c^	0.031

Note: Values in the same row with different superscript letters are significantly different (*p* < 0.05). Values sharing the same letter within a row are not significantly different (*p* > 0.05).

## Data Availability

The raw data supporting the conclusions of this article will be made available by the authors on request.
